# Retrospective Study on Ganglionic and Nerve Block Series as Therapeutic Option for Chronic Pain Patients with Refractory Neuropathic Pain

**DOI:** 10.1155/2020/6042941

**Published:** 2020-07-25

**Authors:** J. D. Gerken, T. Fritzsche, C. Denke, M. Schäfer, S. Tafelski

**Affiliations:** Department of Anaesthesiology and Operative Intensive Care, Charité–Universitätsmedizin Berlin, Campus Charité Mitte and Campus Virchow-Klinikum, Charitéplatz 1, Berlin 10117, Germany

## Abstract

**Objective:**

Current recommendations controversially discuss local infiltration techniques as specific treatment for refractory pain syndromes. Evidence of effectiveness remains inconclusive and local infiltration series are discussed as a therapeutic option in patients not responding to standard therapy. The aim of this study was to investigate the effectiveness of infiltration series with techniques such as sphenopalatine ganglion (SPG) block and ganglionic local opioid analgesia (GLOA) for the treatment of neuropathic pain in the head and neck area in a selected patient group.

**Methods:**

In a retrospective clinical study, 4960 cases presenting to our university hospital outpatient pain clinic between 2009 and 2016 were screened. Altogether, 83 patients with neuropathic pain syndromes receiving local infiltration series were included. Numeric rating scale (NRS) scores before, during, and after infiltration series, comorbidity, and psychological assessment were evaluated.

**Results:**

Maximum NRS before infiltration series was median 9 (IQR 8–10). During infiltration series, maximum NRS was reduced by mean 3.2 points (SD 3.3, *p* < 0.001) equaling a pain reduction of 41.0% (SD 40.4%). With infiltration series, mean pain reduction of at least 30% or 50% NRS was achieved in 54.2% or 44.6% of cases, respectively. In six percent of patients, increased pain intensity was noted. Initial improvement after the first infiltration was strongly associated with overall improvement throughout the series.

**Conclusion:**

This study suggests a beneficial effect of local infiltration series as a treatment option for refractory neuropathic pain syndromes in the context of a multimodal approach. This effect is both significant and clinically relevant and therefore highlights the need for further randomized controlled trials.

## 1. Introduction

The overall prevalence of chronic pain is estimated to be about 19% in Europe [1, 2]. Worldwide, the estimated neuropathic pain in the general population is around ten percent [3]. Particularly, pain in the head and neck area may be debilitating for patients [4]. Pain syndromes such as trigeminal neuralgia (TGN) and persistent idiopathic facial pain (PIFP) often require a multidisciplinary approach [5, 6]. Management can be challenging as patients sometimes do not respond adequately to noninvasive treatment [7]. Therefore, interventional procedures may be considered, when pain persists following the completion of conservative therapy [8, 9].

Feasible techniques in the head and neck area are infiltrations at the sphenopalatine ganglion (SPG), the superior cervical ganglion, the stellate ganglion, and single nerve blocks of the occipital nerve and branches of the trigeminal nerve [10, 11].

Since its first description in 1908, the sphenopalatine ganglion (SPG) block has been discussed in the literature [12]. Recently, an fMRI study showed changes in resting state functional connectivity after SPG block treatment [13]. The literature focuses mostly on SPG block for trigeminal autonomic pain syndromes [14, 15]. Nevertheless, the use in other pain syndromes such as TGN, PIFP, status migrainosus, and postdural puncture headache are described as well [8, 16–19]. The technique appears to be safe and well tolerated [19, 20].

Ganglion local opioid analgesia (GLOA) was first introduced by Mays et al. [21], although the term itself developed later [22]. It describes the infiltration at the superior cervical ganglion, the stellate ganglion, and sometimes even the SPG [23–25]. GLOA has been used as a treatment option for neuropathic and sympathetically mediated pain syndromes of the head, face, and upper extremity [26]. When using the term GLOA, we refer to infiltrations at the superior cervical ganglion as defined by Knolle and Kress [27].

Although different indications are discussed, blockade of the stellate ganglion (SB) is mostly used for Complex Regional Pain Syndrome (CRPS) at the upper extremities [28] as sympathetic nerves pass through the stellate ganglion innervating the head, neck, and upper extremities [29]. In spite of increased evidence for the effectiveness of SB since the beginning of the 2000s [28], the exact role of the sympathetic nervous system in both the development of neuropathic pain and its treatment remains unclear [29, 30].

Peripheral nerve blocks can be helpful in cranial neuralgias such as TGN and glossopharyngeal and occipital neuralgias [31]. Although evidence from clinical trials for efficacy and tolerability is inconclusive [7], an infiltration series as a treatment option for neuropathic pain is used in clinical practice [32].

The reason to use an infiltration series and not a single infiltration is mostly based on historical development of clinical practice [33]. Until now, there is no evidence-based recommendation regarding the number and frequency of infiltrations [33, 34].

Considering this background, this study was performed to investigate the course of pain and pain relief, the associated symptoms, and side effects during the administration of a local infiltration series in patients with refractory neuropathic pain of the head and neck region.

## 2. Materials and Methods

This retrospective study was conducted at the outpatient pain center of the Charité University Hospital, Berlin, Campus Virchow-Klinikum. The department provides clinical care for chronic pain patients and is run by a team of pain specialists, psychotherapists, and trained pain nurses. All cases treated in the department presenting from January 2009 to June 2016 were screened for eligibility. Inclusion criteria were adult patients receiving local infiltration series for treatment of refractory neuropathic pain syndromes (e.g., TGN and trigeminal post-herpetic neuralgia) [35, 36] or pain syndromes with neuropathic characteristics in the head and neck area (e.g., PIFP). Exclusion criteria were missing data files, cases with insufficient documentation, patients without infiltration series in head and neck area, or a different type of pain syndrome (e.g., CRPS). Cases fulfilling the criteria were included for analysis. All infiltration series were embedded in multimodal therapy concepts following the current recommendations [37, 38]. The study was registered on ClinicalTrials.gov with the identifier NCT03066037. The study was approved by the Charité Ethical Committee (EA4/107/16).

### 2.1. Assessment of Pain and the Questionnaires

All patients' contacts in the department were documented in the electronic and paper-based patient filing system. For patients receiving infiltrations, a specific documentation protocol was used. Protocols contain NRS at rest, under stress, before and at 1, 6, and 24 hours after infiltration on an 11-point Likert scale (0–10). A self—assessment of pain reduction and the infiltration's burden (1 = extreme, 2 = severe, 3 = little, and 4 = very little) was assessed on a 4-point Likert scale.

Additional to these infiltration protocols, the daily form of the validated German Pain Questionnaire adopting the 2007 version [39] was used. One question describes average pain; another question describes maximum pain on an 11-point Likert scale (0–10). A different question assesses the endurance of pain on a 4-point scale (1 = not applicable, I have no pain, 2 = I can tolerate it well, 3 = I can just tolerate it, and 4 = I can tolerate it badly). Two questions focus on problems regarding the quality of life (QOL) as suggested by Barker [40]: one examines the impairment in daily activities such as labor, domestic work, and leisure time on an 11-point Likert scale (0–10). A further question describes mental distress on an 11-point Likert scale (0–10).

### 2.2. The Infiltration Technique

Patients were scheduled for the local infiltration series depending on the clinical judgement of the attending pain specialists. The infiltration series were scheduled for ten consecutive infiltrations within three weeks, but the final decision for each infiltration was based on the clinical outcome.

The SPG blockade is based on an injection with local anesthetic agents (2-3 ml bupivacaine 0.25%) and opioid (0, 03 mg buprenorphine) with or without an adjunctive corticoid close to the ganglion (Figure S1). The use of buprenorphine in peripheral nerve blocks has been shown to improve analgesia [41]. We used an infrazygomatic approach based on the standard protocols [11, 42].

To perform a ganglionic local opioid analgesia (GLOA), lipophilic opioids and local anesthetic agents (5 ml 0.5% bupivacaine and 0.03 mg buprenorphine) are injected close to paravertebral and cervical ganglions (Figure S2). We used the anterior paratracheal approach at the C6 level to target the stellate ganglion blockade (Figure S3). Additionally, all single nerves can be targeted with local anesthetic injections, for example, the occipital nerves (Figure S4) and the distal trigeminal nerve branches (Figure S5) [43]. Further details of the techniques can be found in the supplementary materials (Figures S1–S5).

### 2.3. Statistics

All statistical analyses were performed using SPSS 25 and G^∗^Power 3.1.9.6 (post hoc power). Descriptive data was summarized using mean and standard deviation or median and range depending on scale level and distribution. For analysis of statistical significance, NRS scores (0–10) were explored using the exact Wilcoxon signed-rank test for paired data. To analyze independent groups, the Mann–Whitney test was applied. To examine the time point of successful pain reduction, the Kaplan–Meier analysis was performed. For analysis of the correlation between the initial and the final response to infiltration, an exact Chi-square test was performed. To analyze differences in percentage of NRS reduction throughout series by subgroups, the Kruskal–Wallis test was performed. For examination of the response rate between subgroups, the Fisher exact test was performed. All statistical significance tests used a two-sided alpha level of <5% and were intended as exploratory data analysis.

Like previous studies, we defined nonresponders as patients with less than 30% pain reduction and responders as patients with a pain reduction of at least 30% [44]. As additional analysis, we repeated responder analyses with a threshold of 50% pain reduction. To analyze factors associated with responders, we performed univariate and multivariate logistic regression analyses. Based on univariate regression, factors meaningfully associated with the clinical response on an alpha level of at least 10% entered the multivariate regression analysis. This first step was entering the variables sex, age, use of corticosteroids during infiltration, months of preceding pain until the first infiltration, comedication (opioids, antidepressants, and antiepileptics), improvement in maximum NRS scores after the first infiltration, and also improvement in maximum NRS after the first two infiltrations. To assess the calibration of regression analysis, the Hosmer–Lemeshow test was performed. The effect size was examined using Cohen's *f* [45].

## 3. Results and Discussion

**Figure 1:**
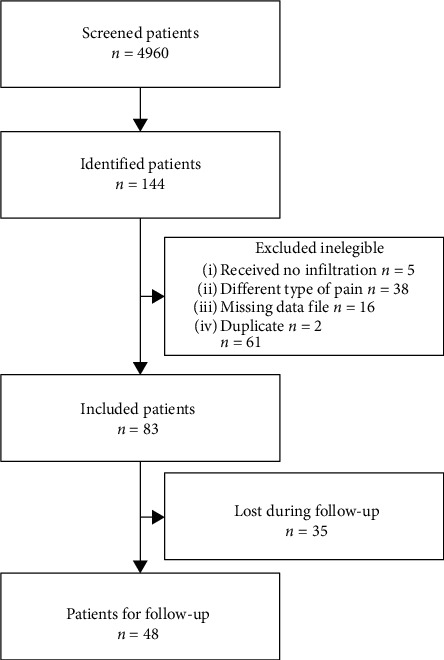
STROBE chart indicating the process of patient recruitment for this retrospective study.

In total, 4960 cases that presented to the pain outpatient center of the Charité University Hospital were screened. Most of these patients did not receive any invasive treatments. For the purpose of this study, patients with refractory pain in the head and neck area scheduled for the local infiltration series between January 2009 and July 2016 were investigated. We identified 144 patients fulfilling the inclusion criteria. After excluding 61 ineligible patients, *N* = 83 patients were included into further analysis (Figure 1). With this subgroup of patients, the mean patient age was 60.4 years (SD ± 15.7) and 50 patients (60.2%) were females. The median time between the onset of pain until the first infiltration was 19 months (IQR 4–65).

### 3.1. Patients' Conditions

Most patients suffered from either trigeminal neuralgia, trigeminal postherpetic neuralgia, or persistent idiopathic facial pain (PIFP) (Table 1). Psychiatric preexisting conditions were present in 23 patients (27.7%) and depression was present in 14 patients (16.9%). Patients were treated with different analgesic and coanalgesic drugs as given in Table 1.

### 3.2. The Blockade Technique

Most patients received a blockade at the sphenopalatine ganglion (*N* = 60, 72.3%) as the main infiltration site. GLOA infiltration was applied in *N* = 12 patients (14.5%) and infiltrations at the stellate ganglion in *N* = 6 (7.2%). Other infiltration sites were the branches of the trigeminal nerve (V1, *N* = 2 patients, 2.4%; V2, *N* = 1, 1.2%) and the major (*N* = 1, 1.2%) and minor occipital nerve (*N* = 1, 1.2%).

### 3.3. Change in Pain: Short-Term Effectiveness

The maximum NRS score leading to the decision to perform the local infiltration series was a median of 9 (IQR 8–10, complete data for *N* = 81 patients). A mean reduction of maximum NRS scores during the course of infiltrations was 3.2 (SD ± 3.3); the median was 3 (IQR 0–6; *p* < 0.001, *N* = 75 patients). This equals a relative average reduction of maximum NRS scores of 40.9% (SD ± 40.4%); the median was 44.4% (IQR 0%–70%).

**Figure 2:**
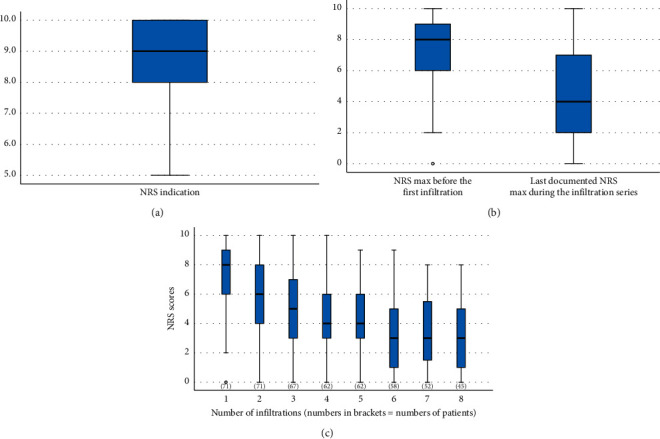
NRS indication, NRS beginning and ending, and NRS during the course of infiltration. (a) A boxplot showing the NRS score for the indication of infiltration series. *N* = 81, mean = 8.65 (SD ± 1.61), and median = 9 (IQR 8,10). (b) Two boxplots comparing the NRS score for maximum pain before the first infiltration and at the ending of infiltration series. A significant NRS reduction was achieved, *p* < 0.001. (c) This graphic shows the NRS scores during the infiltration series until data from at least 50% of initially treated patients was available. Numbers in brackets indicate the numbers of patients reporting NRS scores at that time point.

**Figure 3:**
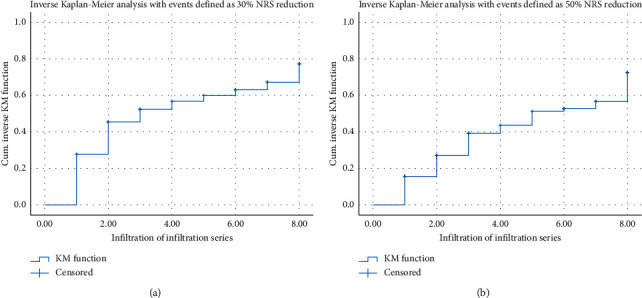
Inverse Kaplan–Meier analysis. (a) Inverse Kaplan–Meier curve with events defined as accomplishment of 30% reduction of initial NRS score. *N* = 83; event *N* = 55 (66.3%). (b) Inverse Kaplan–Meier curve with events defined as accomplishment of 50% reduction of the initial NRS score. *N* = 83; event *N* = 49 (59%).

A reduction of at least 30% of the initial maximum NRS compared to the last documented maximum pain was achieved in 45 patients (54.2%), a 50% reduction in 37 patients (44.6%). Worsening of pain was noted in five patients (6%). The mean increase of maximum NRS in these five patients throughout the series was 1.8 (SD ± 0.83). Numeric rating scale scores during the course of infiltration are presented in Figure 2. Additionally, inverse Kaplan–Meier analysis was conducted to visualize the response to treatment on a 30%- and 50%-level over time (Figure 3).

### 3.4. Burden of the Block Series

During the first infiltration series, patients commonly tolerated the infiltrations well. Categorized burden showed 79.2% of patients reporting little (46.2%) to very little burden (33%). Severe (16.3%) and extreme burden (4.4%) were rarely reported.

### 3.5. Change in Pain: Long-Term Effectiveness

For 48 patients, data from the German Pain Questionnaire was available for a follow-up time point, which was a median of two months (IQR 1–3 months) after the infiltration series.

Evaluating the maximum NRS at the beginning of the infiltration series and the maximum pain ratings at follow-up time point, there was an improvement over time, though not formally statistically significant (*N* = 76, median 8 (IQR 6–9); follow-up *N* = 44, median 6 (IQR 3.3–8), and *p*=0.05) (Figure S6).

Compared with preinfiltration status, patients reported statistically significant improvements of endurance of pain (before median 3 (IQR 3-4); after median 3 (IQR 2-3), *p*=0.023), impact of daily activities (before median 5 (IQR 4–8); after median 4 (IQR 2–7), *p*=0.046), and mental constitution (before median 6 (IQR 5–8); after median 5 (IQR 2.5–7.5), *p*=0.002).

### 3.6. Identification of Responders: Prediction Models

Responders were defined as patients with maximum NRS scores decreasing by at least 30% during treatment. This analysis was repeated for patients with at least a 50% response.

In univariate logistic regression models, variables with potential association with clinical response were included. In the final model, identified relevant variables predicting response were age and relevant improvement in maximum NRS scores after the first two infiltrations (Table 2). Notably, the variable with thehighest predictive value was response after the first infiltration with an OR of 4.833 (95% CI 1.562–14.955). The same approach was used to address a pain reduction of at least 50%. Identified variables associated with a 50% response were age and months with preceding pain until first infiltration, whereas improvement in maximum NRS scores after the first two infiltrations showed a nonsignificant trend (Table 2).

### 3.7. Subgroup Analysis

To further explore clinical subgroups, patients with three dominant pain syndromes in this study population, trigeminal neuralgia, trigeminal postherpetic neuralgia, and persistent idiopathic facial pain (PIFP), were analyzed separately in Table 3.

For patients with trigeminal neuralgia (*n* = 38), clinically relevant NRS reduction of 30% was achieved in 22 cases (51.2%) and a reduction of 50% in 19 cases (44.2%), respectively. In patients with trigeminal postherpetic neuralgia (*n* = 13), a reduction of at least 30% of initial maximum NRS compared to last documented maximum pain was noted in 11 patients (84.6%) and a 50% reduction in eight patients (61.5%). In the PIFP subgroup (*n* = 12), a reduction of 30% NRS throughout series was achieved in 5 cases (35.7%) and a reduction of 50% in 4 cases (28.6%), respectively. Differences in response rates both for 30% reduction (*p*=0.081) and 50% reduction (*p*=0.387) were not statistically significant (Figure S7).

## 4. Discussion

The main findings of this observational study were that (1) the local infiltration series resulted in a meaningful clinical pain reduction of 30% in notably 54% of patients (and 50% pain reduction in 45% of patients); (2) the follow-up patients reported a lasting reduction of pain NRS, along with (3) improvements in pain-related psychometrics. Finally, we were able to identify the variables age, months with preceding pain until the first visit at the outpatient pain center, and improvement in pain NRS scores after the first two infiltrations as predicting factors for clinical response of infiltration series.

Although facial pain is a rare condition with an overall incidence rate of 38.7 per 100,000 patient years, it can have a considerable impact on the patients' quality of life [47]. Refractory pain syndromes are especially difficult to treat and often require a multidisciplinary approach [5], which may include interventional procedures [4, 8].

In patients with neuropathic pain who fail to respond adequately to pharmacological management, the consideration of nonpharmacological therapies is recommended [48]. Invasive treatments are thought to be part of a more comprehensive approach including pharmacological and nonpharmacological, noninterventional treatments [7]. Interventions like infiltrations could alleviate pain in conditions such as TN [49]. Therefore, it is possible to bridge the time gap until long-lasting multimodal treatment can be effective.

There are different approaches to access the sphenopalatine ganglion. Blocks at the sphenopalatine ganglion have been both used with regional placement of local anesthetic through the nose either via soaked cotton tips [12, 50] or placement tools [51] or via different approaches for injections. Ruskin favors the transoral approach [52, 53], while Devoghel points out the risk of this pathway and prefers the suprazygomatic way [14]. We used the infrazygomatic approach, which is easy to target and appears to be safe. A recent imaging study questions whether the local anesthetic drug reaches its goal in the widely used transnasal approach [54]. Due to its effect on the parasympathetic, sympathetic, and sensory nervous system [50] and because of its anatomical connections and role in the trigeminoautonomic reflex [20], the SPG has been the focus of physicians treating pain in the head and neck area. It seems to be important in pain syndromes such as TN, PIFP, and herpes infections [19] and is used for the treatment of status migrainosus [18]. Although employed for various pain syndromes, most of the studies regarding SPG are anecdotal and remain controversial [19].

Regarding GLOA, a previous study shows that its treatment had an adjunctive beneficial effect in carbamazepine-treated patients with trigeminal neuralgia [55]. In a placebo‐controlled double-blind study with 14 patients, Spacek et al. found significant lower NRS values after crossover to the nonplacebo group [56]. Elsner et al. showed a similar beneficial effect of GLOA in a comparable patient group [44]. The superior cervical ganglion, which is targeted by the GLOA, is part of the cervical sympathetic trunk [57] and consequently part of the sympathetic nervous system (SNS) [58].

As there is no communication between autonomic postganglionic sympathetic and parasympathetic neurons with afferent neurons under physiological conditions [59], the role of the SNS in pain is under debate. Nevertheless, under pathophysiological conditions, sympathetic postganglionic neurons may be involved in the generation of pain [59, 60]. It is believed that there is a connection between the SNS and painful peripheral neuropathy [61, 62]. The SNS might regulate neuroactive molecules, immune cells, and peripheral sensitized nociceptors and therefore have an influence on pain modulation [63]. Hence, sympathetically maintained pain could be treated with nerve blocks of the SNS like GLOA [64].

The SNS is targeted in the SB as well: sympathetic nerves passing through the stellate ganglion innervate the head, neck, and upper extremities [29] and its blocks are used in cases of facial pain. Theories of its mechanism involve relief of vascular spasms by blocking sympathetic fibers and thus increasing the blood supply of the brain [29, 65]. Another theory is based on the blocks' sedative effect [66].

The block of the greater occipital nerve (GON) is thought to be an effective treatment for acute migraine [67]. Furthermore, a recent case report describes its use in a patient presenting with persistent headaches following accidental dural puncture [68].

To compare different therapy options, a definition of successful treatment is needed. Although widely used, a definition of the success of nerve blocks is still not established. Generally, a relevant NRS reduction of 30% of initial NRS represents a clinically important difference [69]. Elsner et al. defined an NRS reduction of 30% as satisfying, 50% as good, and 70% as very good [44]. In a survey, German pain therapists estimated that 55% of patients treated in a pain center achieve a successful reduction of pain intensity [32].

Subgroup analyses of the major clinical entities included trigeminal neuralgia, postherpetic neuralgia, and persistent idiopathic facial pain (PIFP). We observed a nonsignificant difference between the response rates to treatment. However, for the limited subgroup of PIFP patients, treatment benefit appeared numerically smaller. In contrast, patients with trigeminal neuralgia and trigeminal postherpetic neuralgia were comparable in terms of treatment response, which may be of relevance in planning prospective trials.

Repetitive blocks are frequently used [32]. One reason is this intention to break the pain cycle by effecting a modulation of autonomic pathways [70]. There is no established number of infiltrations during the series. Repetitions with 3, 6, or 10 infiltrations per series are common [32]; recent study protocols use up to 12 repetitions [51, 71].

Our results show an overall improvement over the first 6 infiltrations. Together with our clinical experience, we recommend at least 6 scheduled infiltrations in a context-sensitive treatment. Notably, our results show that an improvement of NRS in the first and even stronger improvement in the first two infiltrations have a significant predictive value on clinical response. Therefore, the first two infiltrations could be used to identify patients with a higher likelihood to benefit from the treatment. This is relevant since it could prevent performing unnecessary interventions in patients who are unlikely to improve.

Invasive procedures as investigated in this study have enhanced placebo effects [72, 73]. Particularly, physical placebo interventions and patient-involved outcomes such as NRS can especially show the effects of placebo interventions [74]. In a systematic review of migraine prophylaxis, Meissner et al. discussed that more invasive placebo treatments had a stronger effect than less invasive ones. A stronger reduction of migraine frequency was found in sham acupuncture (proportion of responders, 0.38 [95% CI, 0.30–0.47]) and sham surgery (0.58 [0.37–0.77]) than in oral pharmacological placebos (0.22 [0.17–0.28]) [75].

Nevertheless, the effect of placebo interventions on pain is very variable. Hrobjartsson and Gotzsche identified seven placebo interventions on pain trials which were divided into two subgroups: four German acupuncture trials with a pooled effect of standardized mean difference (SMD) −0.68 (−0.85 to −0.50) and three other trials with an effect of SMD −0.13 (−0.28 to 0.03), which equals an effect on pain on a 100 mm visual analogue scale of 16 mm and 3 mm, respectively. Thus, the placebo effect on reported pain ranges from clinically important to irrelevant [74].

In our study, we could show a significant and clinically relevant reduction of NRS. This goes along with findings from a similar study about GLOA as a local infiltration technique [44]. Furthermore, we could detect an improvement in important psychosocial scores such as the impact on daily activities and patient's mental constitution. This is relevant, since neuropathic pain is associated with important impairments of a broad spectrum of health-related quality-of-life domains [76–78].

### 4.1. Limitations

However, this study has limitations. The retrospective design limits the interpretation of the results. Furthermore, it is not possible to distinguish between the effects of blocks, the placebo effect, other treatments and the natural course of pain syndromes. In addition, there could be a selection bias regarding the follow-up group, since patients without pain are more likely not to visit our outpatient pain clinic. Moreover, the subgroup of patients with PIFP could have a lower response rate to treatment and thus estimated that the overall study response rates could be underestimated in their assessment. Additionally, there is very limited literature regarding head and neck nerve blocks and no standardization of its performance [43]. Particularly, the techniques used for SPG block and GLOA vary widely between different authors.

In this investigation with repetitive data collections, we could not control for all factors, e.g., intraindividual variance of patients. For example, for plain NRS reduction of 3.2 points in pain intensity over the course of treatment, we achieved a power of 99.9% (*p* = 0.001), which does suggest a low probability of bias regarding the intraindividual variation. The latter may also be an important piece of information in designing prospective trials. Although there are potent correction methods for multiple comparisons, this study is exploratory with a nonconfirmatory design. In such studies, conservative procedures like Bonferroni's correction may further increase the risk of type 2 errors [79].

### 4.2. Clinical Significance

Local infiltration series are used in patients with refractory neuropathic pain syndromes despite lacking evidence. Our study provides data on effectiveness and the burden of local infiltration techniques in patients not responding to standard therapy. This is important, since randomized—controlled trials are ethically difficult to perform in invasive procedures. Furthermore, we have evaluated factors to predict response to therapy to guide patient—management during the infiltration series.

## 5. Conclusions

In summary, this retrospective analysis demonstrates a beneficial effect of local infiltration techniques as a treatment option for refractory pain syndromes. The effects of the treatment with the infiltration series are significant and appear clinically relevant as therapeutic options are limited in this population [48, 80]. Further studies with prospective, controlled design and long-term data are necessary to validate these findings.

## Figures and Tables

**Table 1:** Basic characteristics for *N* = 83 patients included with refractory neuropathic pain syndromes in the head-neck area.

Variable	N = 83 patients
Female gender *n* (%)	50 (60.2%)
Age in years mean (±SD)	60.4 (±15.7)
Median (25–75 quartile)	57 (51–74)
Duration onset of pain until first infiltration (months) (*N* = 76), median (quartiles)	19 (4–65)
Medication at the beginning of infiltration series *n* in %	
WHO I: *n* (%)	20 (24.1%)
WHO II: *n* (%)	16 (19.3%)
WHO III: *n* (%)	18 (21.7%)
Coanalgesic drugs:	
Antidepressants *n* (%)	65 (78.3%)
Antiepileptics *n* (%)	75 (90.4%)
Other	
Psychiatric pre‐existing condition *n* (%)	23 (27.7%)
Depressions *n* (%)	14 (16.9%)
Infiltration with additional corticosteroid *n* (%)	69 (83.1%)
Neuropathic pain classified following ICHD 3 [46], given in *n* (%)	
Headache or facial pain attributed to other disorders of cranium, neck, eyes, ears, nose, sinuses, teeth, mouth, or other facial or cervical structure, 11.9	5 (6%)
Trigeminal neuralgia, 13.1	43 (51.8%)
Trigeminal postherpetic neuralgia, 13.1.2.2	13 (15.7%)
Persistent idiopathic facial pain (PIFP), 13.12	14 (16.9%)
Others	8 (9.6%)

**Table 2:** Logistic regression analyses.

	Univariate regression analysis		Multivariate regression analysis	
	OR (95% SD)	*p*	OR (95% SD)	*p*
*A*				
Female sex	0.684 (0.261–1.791)	0.440		
Corticoid use during infiltration	1.156 (0.357–3.750)	0.809		
Age	1.045 (1.012–1.080)	0.007	1.052 (1.009–1.098)	0.018
Months in pain until the first visit	1.004 (0.996–1.011)	0.347		
Cotherapy opioids	0.552 (0.215–1.413)	0.215		
Cotherapy antidepressants	1.000 (0.315–3.174)	>0.999		
Cotherapy antiepileptics	1.000 (0.157–6.373)	>0.999		
Improvement after the first infiltration	4.833 (1.562–14.955)	0.006		
Improvement after the first two infiltrations	8.017 (2.431–26.436)	0.001	7.484 (2.089–26.816)	0.002

*B*				
Female sex	0.682 (0.268–1.737)	0.423		
Corticoid use during infiltration	1.378 (0.427–4.46)	0.592		
Age	1.034 (1.004–1.066)	0.029	1.061 (1.016–1.108)	0.008
Months in pain until the first visit	1.007 (0.999–1.015)	0.079	1.011 (1.011–1.021)	0.024
Cotherapy opioids	0.602 (0.238–1.522)	0.284		
Cotherapy antidepressants	0.819 (0.263–2.543)	0.729		
Cotherapy antiepileptics	0.630 (0.099–4.003)	0.624		
Improvement after the first infiltration	2.413 (0.858–6.781)	0.095		
Improvement after the first two infiltrations	3.491 (1.125–10.829)	0.030	3.579 (0.898–14.255)	0.071

Univariate and multivariate logistic regression analyses were used to predict a clinical response of at least 30% (A) and a pain reduction of 50% (B) as dependent variable. Multivariate analysis was performed with variables of at least *p*=0.1 in univariate testing. Hosmer–Lemeshow goodness-of-fit test was sufficiently for multivariate regression model (A: Chi^2^ 12.056, *p*=0.099; *N* = 66; B: Chi^2^ 8.763, *p*=0.363; *N* = 61).

**Table 3:** Pain reduction for study subgroups. Absolute NRS reduction did not differ statistically between groups (*p*=0.276).

	Number of patients	Mean NRS reduction (SD)	Median NRS reduction (IQR)	*p*
Trigeminal neuralgia	38	3.5 (±3.2)	3.5 (0–6.25)	<0.001
Trigeminal postherpetic neuralgia	13	3.6 (±1.8)	3.0 (2–5)	=0.001
PIFP	13	1.6 (±4.3)	0 (0–5)	=0.159

## Data Availability

Data can be accessed upon request by contacting the corresponding author PD Dr. Sascha Tafelski via e-mail: sascha.tafelski@charite.de.

## Supplementary Materials

Supplementary Materials Figure S1: SPGB—patient sits with their head turned away from the injection site. Figure S2: GLOA. Figure S3: stellate ganglion block. Figure S4: trigeminal nerve block. Figure S5: blockade of the greater occipital nerve (GON) and the lower occipital nerve (LON). Figure S6: comparison of NRS for maximum pain between the start and at follow-up. Figure S7: achievement of 30% and 50% NRS reduction throughout series.
