# Supplementary material for: Retrospective Study on Ganglionic and Nerve Block Series as Therapeutic Option for Chronic Pain Patients with Refractory Neuropathic Pain
Source: Pain Res Manag. 2020 Jul 25;2020:6042941. doi: 10.1155/2020/6042941 (PMC7399767; doi:10.1155/2020/6042941)
Supplement: Supplementary Materials — Figure S1: SPGB—patient sits with their head turned away from the injection site. Figure S2: GLOA. Figure S3: stellate ganglion block. Figure S4: trigeminal nerve block. Figure S5: blockade of the greater occipital nerve (GON) and the lower occipital nerve (LON). Figure S6: comparison of NRS for maximum pain between the start and at follow-up. Figure S7: achievement of 30% and 50% NRS reduction throughout series. [file 6042941.f1.pdf]

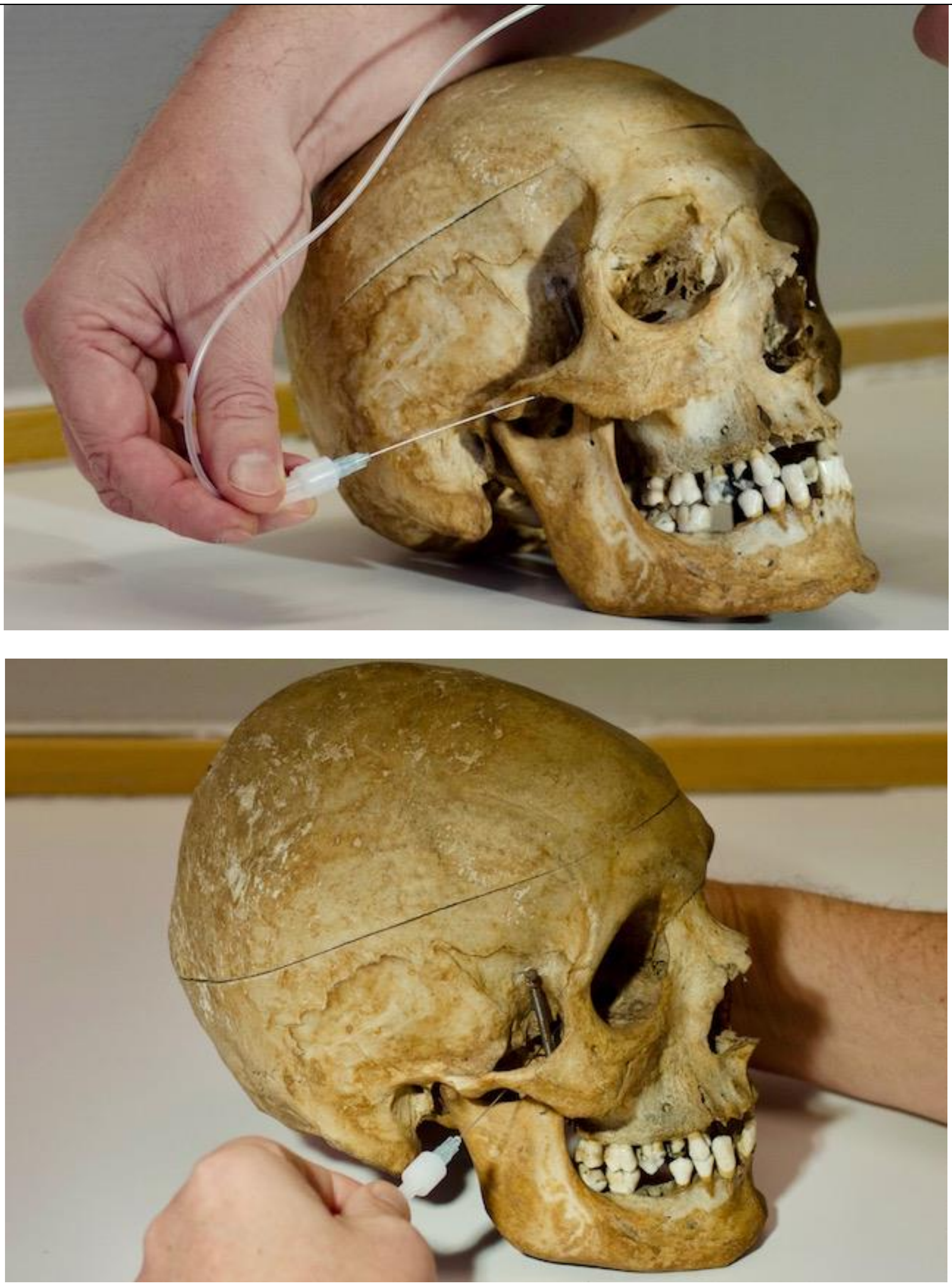

Figure S1: SPGB - Patient sits with their head turned away from the injection site. The skin is pearced by an 8cm long 22G needle at 90 degrees to the skin surface in the middle of the Incisura mandibulae directly underneath the zygomatic arch. At the depth of around 4cm (depending on the patient's anatomy), the needle tip reaches the lateralpterygoid lamina. The needle is then withdrawn to the subcutaneous layer and reincerted ventrocranially for one additional centimeter. If resistance is felt and the needle cannot be inserted further, the needle tip is either in contact with the ptygoid process or at the maxilla. A further change of direction may be indicated. Once, the final position is located, 2-3ml Bupivacain 0.5% and 0.03mg Buprenorphine are injected.

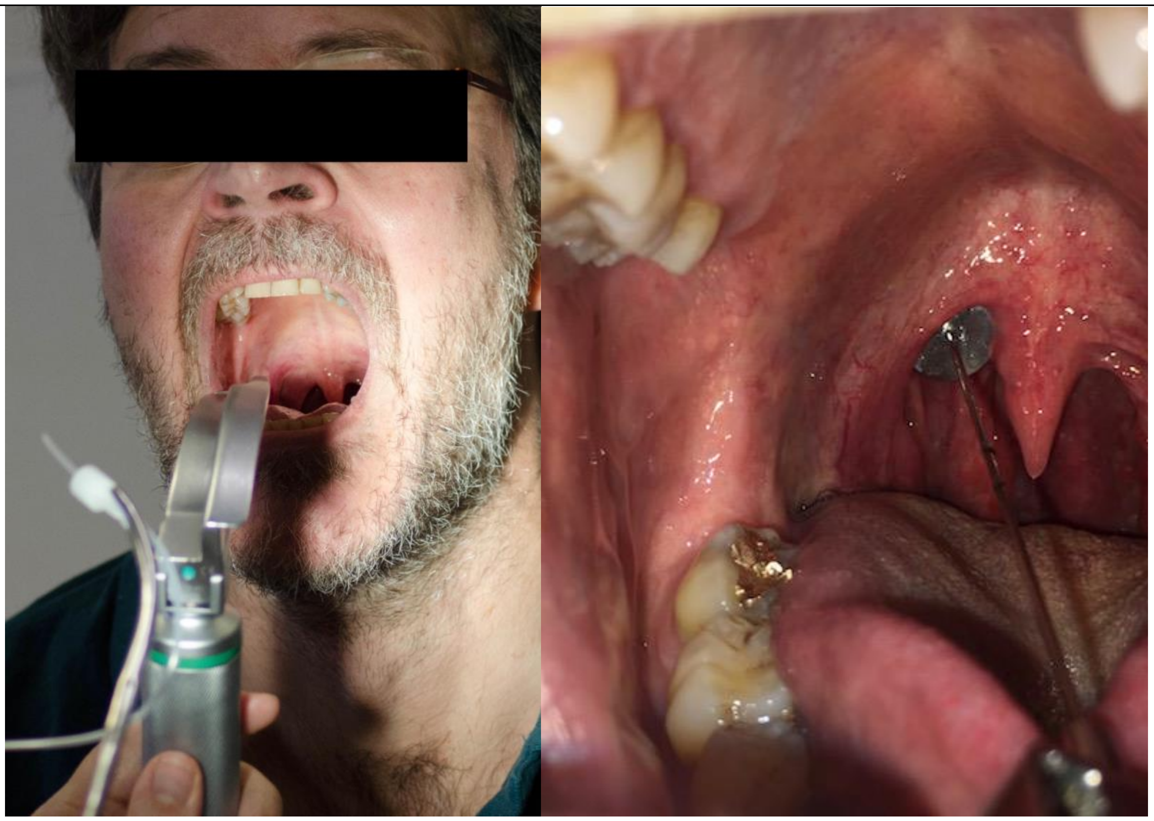

Figure S2: GLOA

The patient sits on a chair with their mouth wide open. Careful insertion of a laryngoscope upside down as a tongue depressor and light source. Intraoral injection using a 25G needle at C2 level for a maximum depth of 10mm retrotonsillary at the lateral pharyngeal wall. A SPROTTE® needle can be used to keep the distance as shown in second picture [78]. If aspiration test was negative, injection of 5ml 0.5% Bupivacain and 0.03mg Buprenorphine.

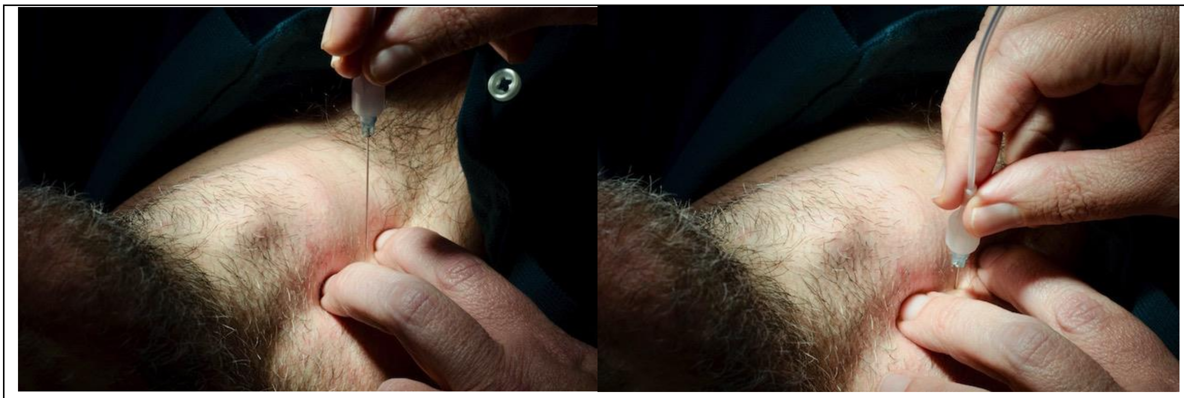

Figure S3: Stellate ganglion block

According to Herget as described by Dosch [10]: The patient lies in the supine position with a firm pad under the shoulders to hyperextend at the cervical spine. The skin is pierced with an 8cm long needle perpendicularly to the skin surface. The injection point lies on the transition from the lower to the middle third, on an auxiliary line between mastoid and sternoclavicular joint. Its position is around 20-30mm lateral from the midline at the sternomastoid muscle's medial margin. At a depth of 60–70mm, the point of the needle reaches the tuberculum caroticum at C6 level. The needle is drawn back for 2mm and aspiration is performed. If the aspiration test was negative, infiltration of 10ml bupivacaine 0.25% can be executed. If the needle lies in the correct position, the patient develops a homolateral Horner's syndrome with ptosis, myosis, and enophthalmos.

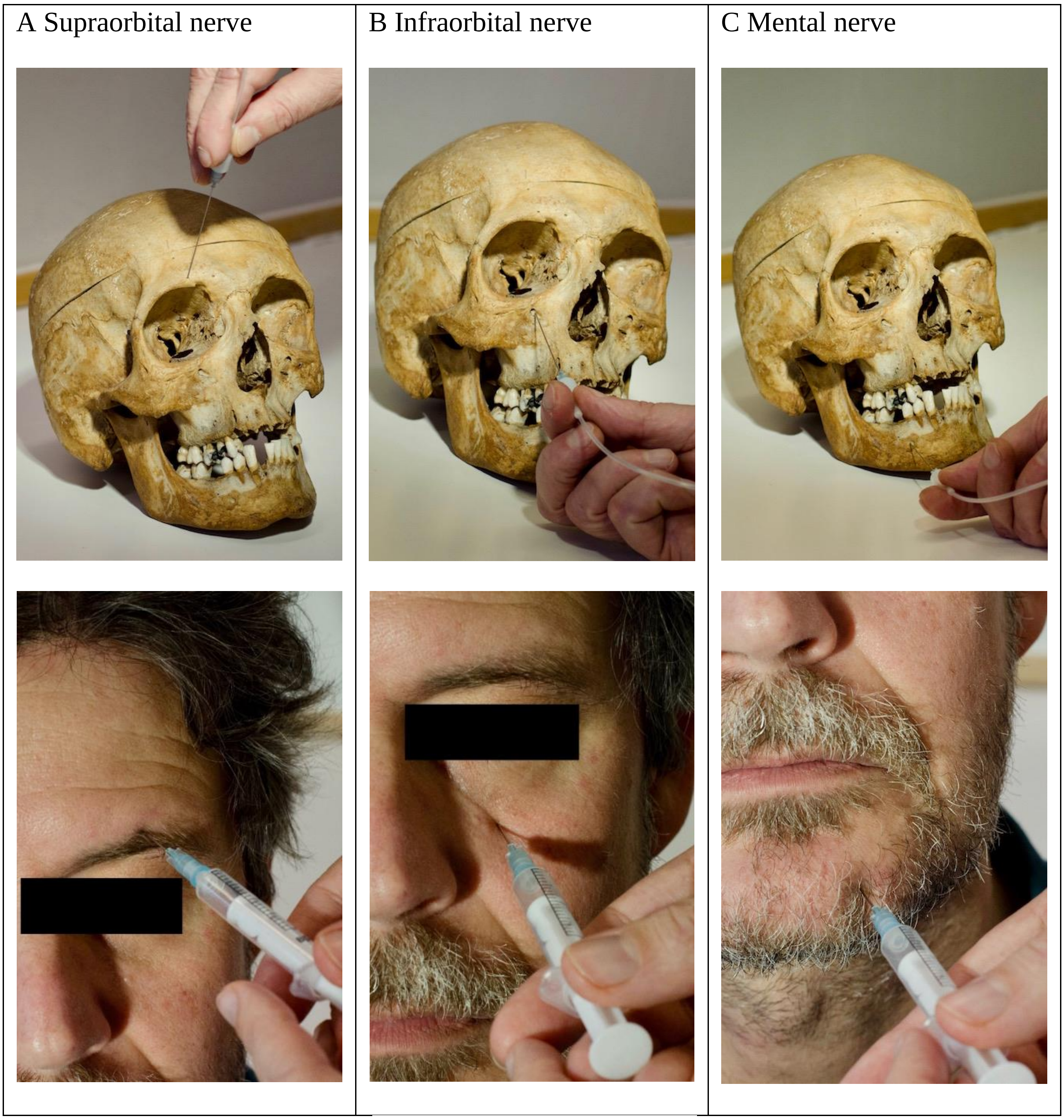

<u>Figure S4:</u> Trigeminal nerve block

A: The patient sits with their head turned away from the injection side, while the supraorbital notch is being palpated. Skin penetration with an 4cm long 25G needle at the level of the notch and advanced medially at 15degrees until it reaches the periosteum. If the aspiration test was negative, infiltration of 2-3 ml bupivacaine 0.5% can be distributed in a fanlike manner [42]. **B:** The patient sits with the head turned to the opposite side. Identification of the infraorbital foramen by palpation. Skin puncture with a 4cm long 25G needle at the level of the notch and advanced medially 15° until it reaches the periosteum. If the aspiration test was negative, infiltration of 2-3 ml bupivacaine 0.5% can be distributed in a fanlike manner [42]. **C:** The patient sits with the head turned to the opposite side. Identification of the mental notch by palpation. The skin puncture with an 4cm long 25G needle at the level of the notch and advanced medially 15° until it approaches the periosteum. If the aspiration test was negative, infiltration of 2-3 ml bupivacaine 0.5% can be distributed in a fanlike manner [42].

A: Greater occipital nerve (GON) | B: Lower occipital nerve (LON)

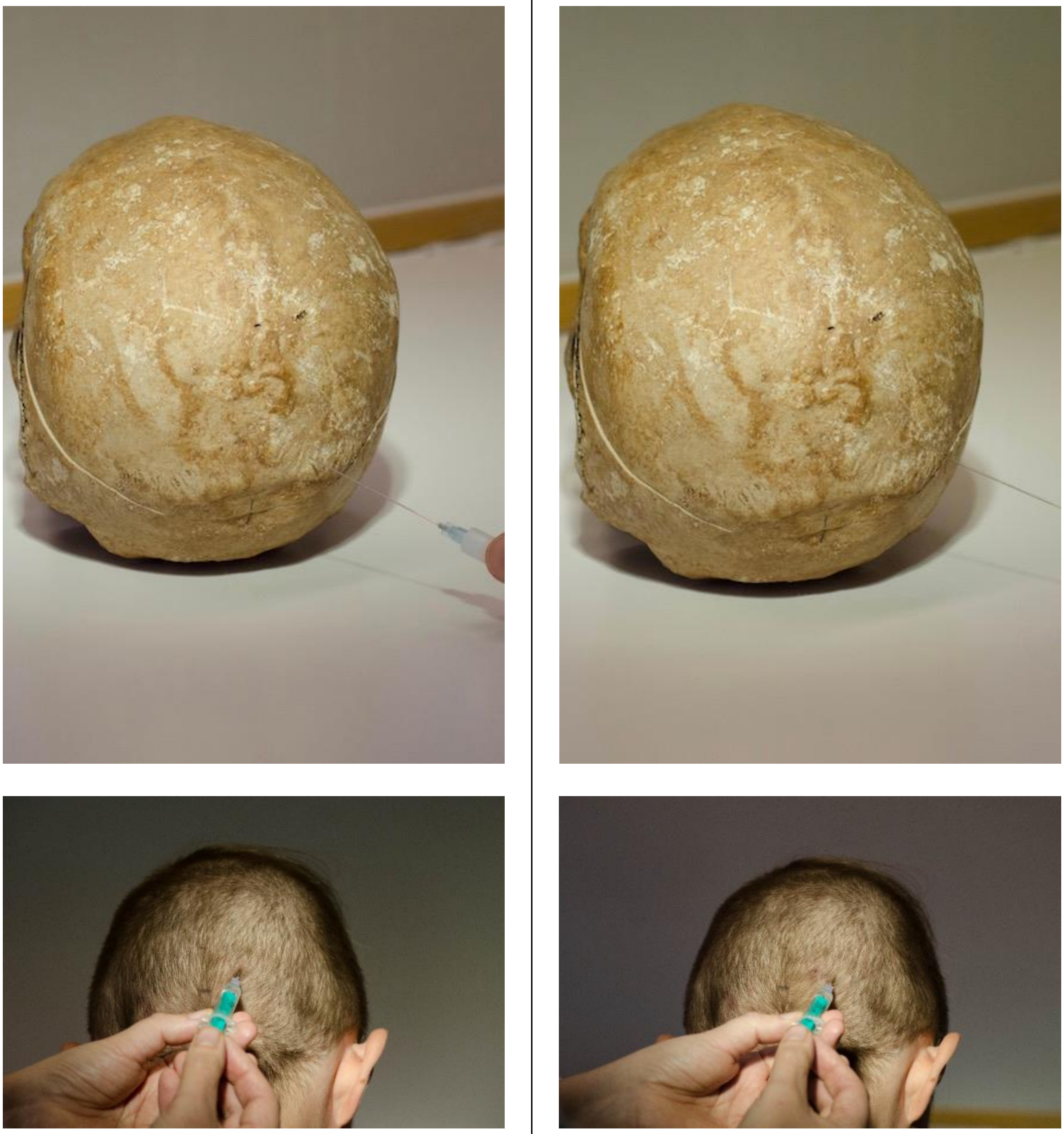

Figure S5: blockade of the Greater occipital nerve (GON) and the Lower occipital nerve (LON).

A: The patient is in a seated position with their head on their chest for the identification of the occipital protuberance. The occipital artery lays about 4cm lateral to the occipital protuberance in direction of the mastoid process. The nerve is often directly medial to the artery - approximately 2.5-3cm lateral to the occipital protuberance. Insertion of the needle in an anterior and slightly superior direction until it approaches the skull bone or the patient reports paresthesia. Withdraw the needle a couple of millimeters. If the aspiration test was negative, infiltration of 2-5 ml bupivacaine 0.5% can be distributed either directly into the site or in a fanlike manner[42]. **B:** Similar blockade technique as GON. The LON is located about 2.5 cm lateral to the occipital artery [42].

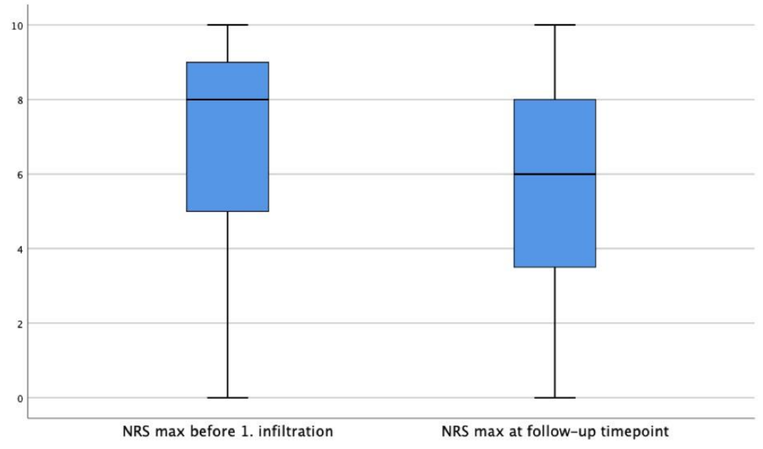

Figure S6: Comparison of NRS for maximum pain between the start and at follow-up. Formally, no significant decrease was noted (before n=76, median= 8 (IQR 6-9); after n= 44, median= 6 (IQR 3.25-8), p = 0.050).

A Achievement of 30% NRS reduction

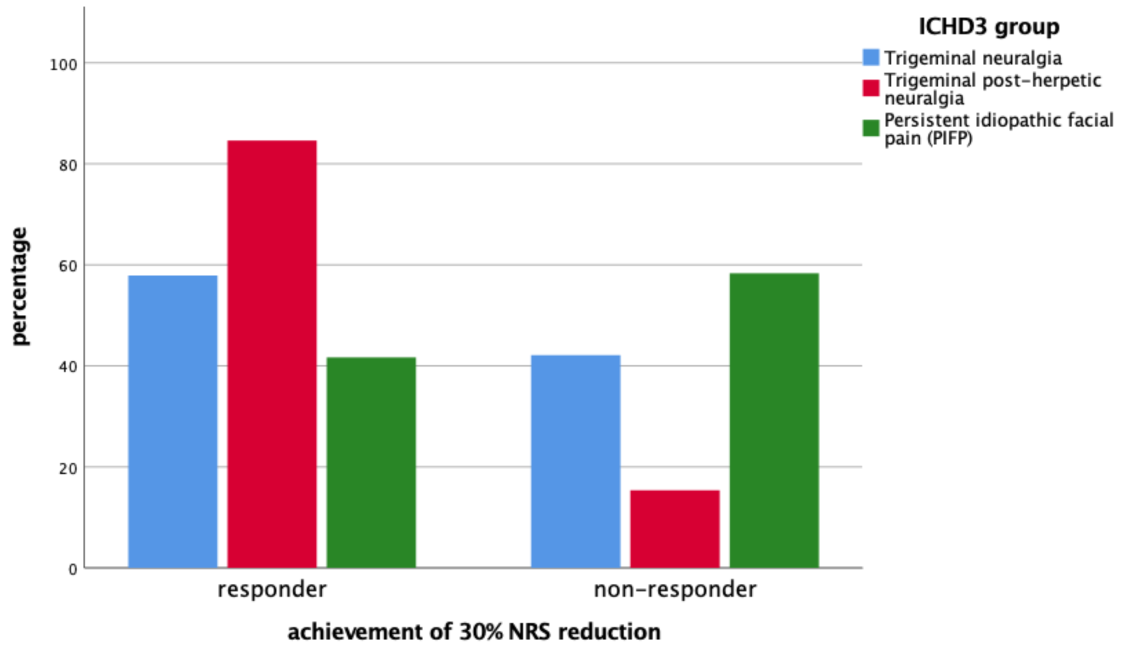

B Achievement of 50% NRS reduction

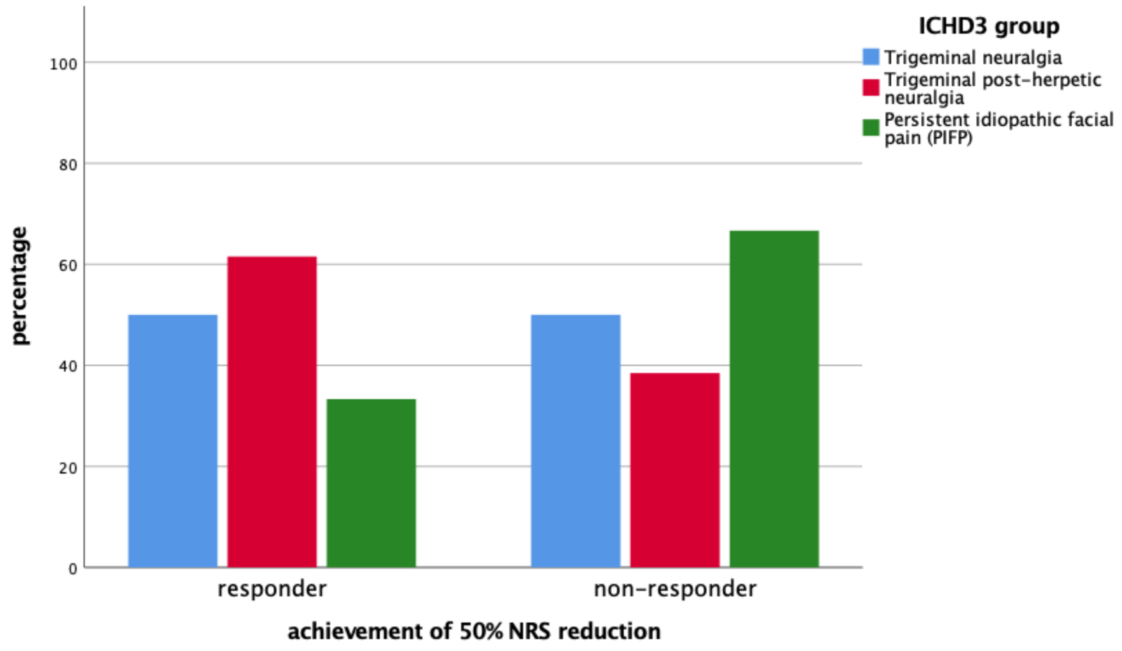

Figure S7: Achievement of 30% and 50% NRS reduction throughout series.

A: Percentage of patients defined as responders with achieved NRS reduction of at least 30% comparing NRS at the beginning and end of the infiltration series divided by their main underlying pathology. A non-significant difference was noted (n=63; p=0.081). **B:** Same analysis as in A with 50% NRS reduction. Differences between subgroups were not significant (n=63; p=0.387).
